# Direct Scaffold-Coupled Electrical Stimulation of Chondrogenic Progenitor Cells through Graphene Foam Bioscaffolds to Control Mechanical Properties of Graphene Foam – Cell Composites

**DOI:** 10.21203/rs.3.rs-5589589/v1

**Published:** 2024-12-24

**Authors:** Mone’t Sawyer, Amevi Semodji, Olivia Nielson, Attila Rektor, Hailey Burgoyne, Michael Eppel, Joshua Eixenberger, Raquel Montenegro-Brown, Miranda L. Nelson, Trevor Lujan, David Estrada

**Affiliations:** Boise State University; Boise State University; University of Idaho; Boise State University; Boise State University; Boise State University; Boise State University; Boise State University; Boise State University; Boise State University; Boise State University

**Keywords:** graphene foam, electrical stimulation, tissue engineering, dynamic mechanical analysis, unconfined compression, correlative microscopy, ATDC5, microcomputed tomography, volumetric analysis

## Abstract

Osteoarthritis, a major global cause of pain and disability, is driven by the irreversible degradation of hyaline cartilage in joints. Cartilage tissue engineering presents a promising therapeutic avenue, but success hinges on replicating the native physiological environment to guide cellular behavior and generate tissue constructs that mimic natural cartilage. Although electrical stimulation has been shown to enhance chondrogenesis and extracellular matrix production in 2D cultures, the mechanisms underlying these effects remain poorly understood, particularly in 3D models. Here, we report that direct scaffold-coupled electrical stimulation applied to 3D graphene foam bioscaffolds significantly enhances the mechanical properties of the resulting graphene foam – cell constructs. Using custom 3D-printed electrical stimulus chambers, we applied biphasic square impulses (20, 40, 60 mVpp at 1 kHz) for 5 minutes daily over 7 days. Stimulation at 60 mVpp increased the steady-state energy dissipation and equilibrium modulus by approximately 65% and 25%, respectively, compared to unstimulated controls, while also yielding the highest cell density among stimulated samples. In addition, our custom chambers facilitated full submersion of the hydrophobic graphene foam in media, leading to enhanced cell attachment and integration across the scaffold surface and within its hollow branches. To assess this cellular integration, we employed co-localized confocal fluorescence microscopy and X-ray microCT imaging enabled by colloidal gold nanoparticle and fluorophore staining, which allowed visualization of cell distribution within the opaque scaffold’s internal structure. These findings highlight the potential of direct scaffold-coupled electrical stimulus to modulate the mechanical properties of engineered tissues and offer new insights into the emergent behavior of cells within conductive 3D bioscaffolds.

## Introduction

Osteoarthritis (OA) is a prevalent musculoskeletal condition caused by articular cartilage (AC) degradation in synovial joints. It ranks as a world-leading cause of pain and disability, currently affecting over 595 million individuals – more than double the 256 million afflicted individuals recorded in 1990^1–3^. The avascularity, low cell density, and lack of innervation in AC hinder its self-healing capabilities, driving the development of innovative strategies for functional restoration^4^. Conventional treatments for OA involve total joint replacement and symptomatic relief through invasive and expensive procedures, such as arthroscopy for cartilage fragment removal and microfracture for cartilage regeneration^5^. Beyond total joint replacement, procedures typically provide only temporary relief, particularly in severe cases, highlighting the need for more effective OA treatments.

Tissue engineering (TE) offers a promising solution for OA, with three-dimensional (3D) porous bioscaffolds playing a role in how cells proliferate and differentiate to form functional chondrogenic tissues^6^. Although several advances have been made in engineering AC, significant challenges persist in translating *in vitro* innovations into effective clinical treatments^7^. AC’s unique structural properties are dynamically regulated by chondrocytes, providing it with a hierarchical organization essential for mechanical stability and load-bearing capacity^8^. Although chondrocytes do not directly contribute to the mechanical properties, they sense and respond to their microenvironment where discrepancies in bioscaffold stiffness can have downstream effects on cellular behavior and subsequent tissue development^9–11^. A wide range of hydrogels, polymers, and their composites have been used for AC TE to replicate the micro-mechanical environment necessary for cartilage tissue growth^12^. However, these scaffolds often fail to match the mechanical properties of native AC. For instance, polymer-based scaffolds typically have compressive moduli between 2 and 50 kPa, which is substantially lower than that of human AC, ranging from 250 to 1800 kPa^8,13,14^. Other materials studied have sufficient mechanical properties but lack a highly porous microstructure, which is essential for cell infiltration and nutrient diffusion^15^. This highlights the need to balance mechanical strength and bioscaffold architecture^11^.

Additionally, externally supplied physical and chemical cues have been used to influence fluid flow, nutrient and ion concentration gradients, anabolic and catabolic activity of extracellular matrix (ECM) components, and synthesis of growth factors^16–19^. Physical stimuli such as mechanical compression, cyclic loading, fluid shear stress, and tensile strain have been widely utilized during culture to simulate the physiological forces experienced by native cartilage^11,20,21^. Chemical cues, including growth factors, hormones, and signaling molecules, bind to receptors and activate signaling pathways that drive proliferation and chondrogenesis. These interactions are necessary to promote the synthesis and organization of cartilage-specific ECM components, which are important for maintaining the engineered tissue’s structural integrity and functionality^22,23^.

**Figure 1:**
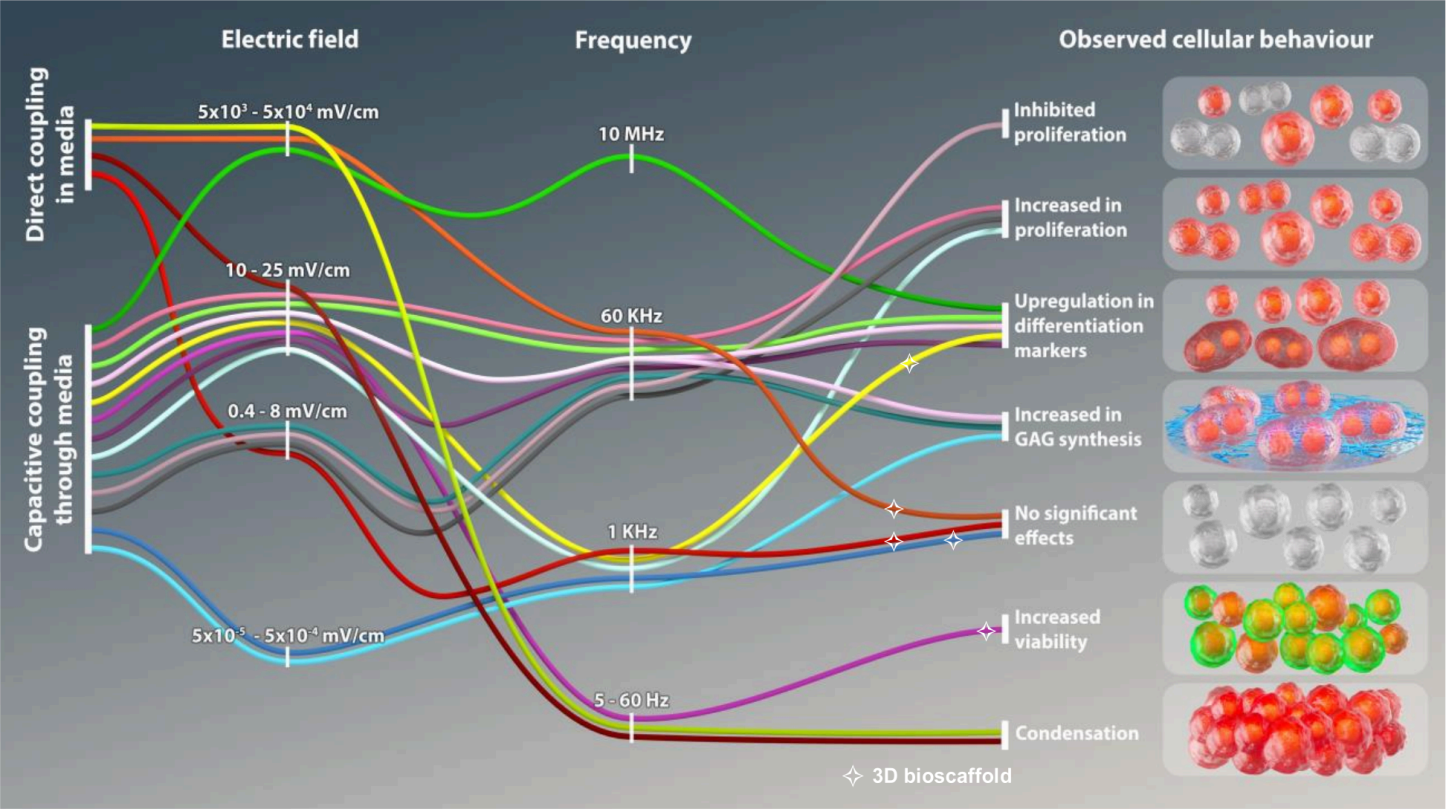
Alluvial diagram illustrating the diverse effects of electrical stimulus parameters on cellular responses in cartilage tissue engineering applications. The diagram visualizes the relationship between the coupling method, electric field strength, frequency, and observed cellular behavior across 16 studies. Studies utilizing 3D bioscaffolds are denoted by a star (✧), highlighting their role in specific coupling conditions. The diagram emphasizes the variability in electrical stimulus protocols and their impact on cellular processes, including proliferation, differentiation, condensation, ECM synthesis, and cell viability. 26,33,34,67,93–101

Recently, *in vitro* electrical stimulus (ES) has opened new avenues for driving cell growth and differentiation. Previous studies show ES can enhance cellular condensation, boost chondrogenic proliferation, and stimulate the synthesis of ECM molecules^24^. For example, high-voltage ES has been shown to induce transmembrane ion transport, leading to intracellular Ca^2+^ flux through voltage-operated calcium channels^25^. In contrast, low-voltage ES has led to cell condensation and upregulation of chondrogenic genes^26,27^. Understanding the precise mechanisms of ES on cells remains challenging due to such inconsistent protocols, including capacitive coupling and direct ES in ionic media (Fig. 1). This lack of standards can complicate linking quantitative electric fields and transmembrane potentials to cellular response^28–31^. Nonetheless, previous studies provide valuable insights and suggest that further investigation into the effects of field dissipation related to electrode distance, thermal heating, and the homogeneity of applied ES could enhance our understanding of the mechanisms driving fundamental cellular behavior. ES using two-dimensional (2D) monolayer studies fostered chondrogenic differentiation and cartilage maturation, yet translating these protocols to 3D tissue applications remains challenging^32^. Recent studies exploring the effects of ES in 3D environments have reported varied outcomes, emphasizing the importance of scaffold properties and ES parameters.

One such study applied capacitive coupling using collagen-elastin based scaffolds, delivering estimated field strengths of 5 × 10^− 5^ mV/cm and 5 × 10^− 4^ mV/cm at a frequency of 1 kHz for 7 days (45 minutes, three times daily)^33^. At 5 × 10^− 5^ mV/cm no significant changes in chondrocyte metabolic activity or gene expression were observed. However, at 5 × 10^− 4^ mV/cm, an increase in glycosaminoglycan (GAG) synthesis was measured, indicating a voltage-dependent effect on gene and protein expression. Achieving these low electric fields required input voltages of 100 mVpp and 1 Vpp, underscoring the challenge of generating effective field strengths in non-conductive scaffolds and the impact of the high resistance of ionic culture media.

A subsequent study developed a custom device with embedded electrodes at the bottom for capacitive coupling, delivering estimated electric field strengths of 2.0–2.5 mV/cm (for a 30 mVpp input at 60 kHz) and 0.32–0.40 mV/cm (for an input of 30 mVpp at 1 kHz)^34^. Their findings revealed a frequency-dependent impact on chondrocyte behavior, with trends indicating reduced collagen type I release and subtle modulation of chondrogenic markers, although no significant differences were observed between the control and stimulated samples. The electric fields demonstrated spatial heterogeneity, with the strongest fields localized to 1 mm above the electrode and diminishing with height, a limitation potentially affecting the efficacy of uniform stimulation. This highlights the challenge of optimizing both field strength and distribution, particularly when scaffold conductivity may constrain effective field application.

An additional study explored the effects of ES on mesenchymal stem cells encapsulated in hyaluronic acid-gelatin injectable hydrogels, applying estimated electric fields of 10 mV/cm at 60 kHz over 21 days (30 minutes, four times daily). Notably, ES significantly enhanced chondrogenic differentiation without the addition of exogeneous growth factors, evidenced by increased expression of SOX9 and aggrecan, alongside a 2.43-fold increase in collagen type II relative to total collagen content^35^.

Results from literature suggest that optimizing the parameters, stimulation mode, and scaffold material are critical for the *in vitro* modulation of chondrocyte metabolism^36^. In this context, graphene foam (GF) bioscaffolds present a promising conductive 3D bioscaffold to apply scaffold-coupled direct ES to adherent cells at low electric fields and probe fundamental insights into the emergent behavior of cells in response to ES for 3D TE applications^37^.

Graphene, an allotrope of carbon, is a 2D, single-atomic layer structure consisting of six bonded sp^2^ carbon atoms arranged in a honeycomb lattice^38^. Due to its planar configuration graphene has high electron mobility at room temperature (> 15,000 cm^2^ V^− 1^ s^−1^), exceptional thermal conductivities (3000–5000 W m^− 1^ K^− 1^), high mechanical strength with a Young’s modulus of 1 TPa, and extraordinary chemical stability^39–42^. These distinctive properties of graphene, as well as its flexibility and biocompatibility, render it a prime candidate as an active bioscaffold, where its electrical characteristics can be harnessed to deliver electrical signals in vitro^43–45^. GF, synthesized through template assisted chemical vapor deposition (CVD), has a 3D architecture made up of interconnected graphene sheets that results in hollow branches connected by node junctions. The CVD technique allows for control over the number of graphene layers and the porosity of the foam, resulting in the ability to tailor GF bioscaffolds to specific TE applications^46^. Due to its 3D structure, GF exhibits high mechanical strength with an elastic modulus ranging from 1.2 to 69.9 GPa^47,48^. GF’s highly porous architecture, coupled with its distinct node shapes, enhance load-bearing capabilities and energy absorption. This interconnected network of branches and node junctions provides a seamless pathway for stress transfer upon mechanical loading making it suitable for applications that require both mechanically robust and flexible bioscaffolds^49,50^. These properties, coupled with its high electrical conductivity (10–1600 S·cm^− 1^) and biocompatibility, make GF an excellent candidate for active bioscaffolds capable of delivering electrical signals *in vitro*^46,51^. While recent studies have probed the effect of ES on stem cell growth and differentiation on planar graphene film bioscaffolds, scaffold-coupled ES applied *in vitro* to cells cultured on GF, has yet to be investigated^52^. ^50^

This study aims to evaluate how scaffold-coupled ES impacts the mechanical properties of GF-cell composites by leveraging the conductive nature of GF to facilitate direct ES to cells embedded within the scaffold. We employed custom 3D-printed ES chambers (ESC) to deliver direct ES to ATDC5 chondrogenic progenitor cells, seeded on GF, and assessed the resultant mechanical behavior with dynamic mechanical analysis (DMA). The ATDC5 model encapsulates major aspects of cartilage biosynthesis and ECM maturation, making it a useful model for investigating mechanisms that regulate chondrogenesis *in vitro*^53,54^. Figure 1 demonstrates that cellular responses to ES vary depending on the mode of application, with capacitive coupling and direct coupling in ionic media often leading to inconsistent outcomes across literature. By applying direct scaffold-coupled ES to cells cultured on GF within ESCs, we aimed to standardize stimulation parameters for consistent and reliable application^55,56^. Our approach demonstrates significant improvements in the mechanical performance of the GF-cell constructs within 14 days of culture, highlighting the potential for accelerated biomechanical enhancement through scaffold-coupled ES.

## Results

### GF Synthesis and Characterization

**Figure 2:**
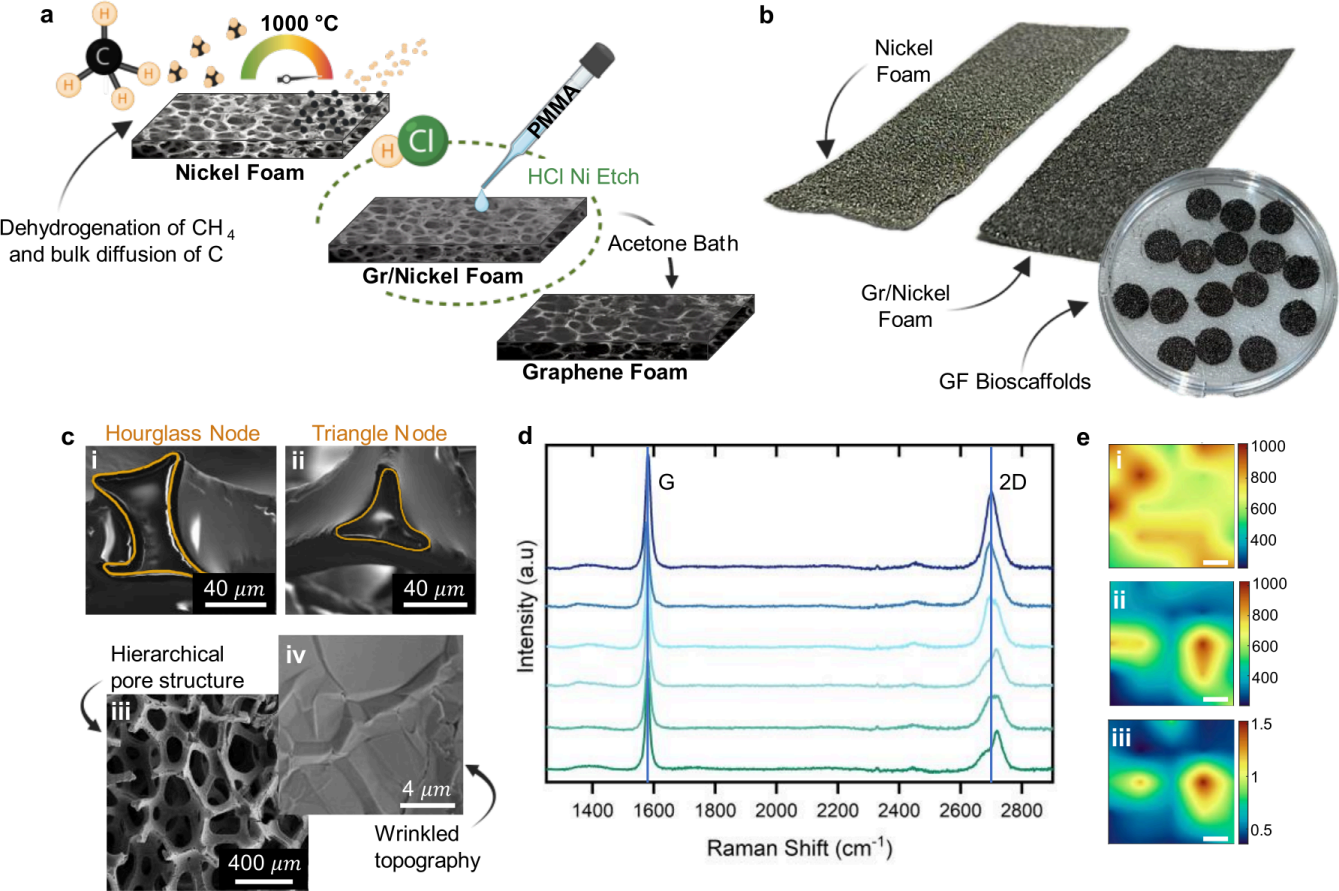
Synthesis and characterization of GF. a) Schematic illustrating the synthesis of CVD GF on a nickel (Ni) foam template. Graphene-coated nickel foam (Gr/Nickel) is coated with polymethyl methacrylate (PMMA) to maintain integrity during Ni etching in 3M HCl. After Ni removal, PMMA is dissolved in an acetone bath, resulting in free-standing GF. b) Macroscale images of Ni foam before and after graphene synthesis, and the subsequent GF bioscaffolds. c) Cross-sectional SEM analysis revealing (i) hourglass and (ii) triangle node junctions where structure thickness is measured and surface SEM images show the (iii) hierarchical porous structure and (iv) wrinkled surface topography of the CVD GF. d) Raman spectra from six different GF samples, displaying the characteristic G-peak (IG at ≈ 1580 cm^−1^) and 2D-peak (I2D at ≈ 2700 cm^−1^), confirming the graphitic structure of GF. e) Raman spatial mapping over a 100*μm*^2^ area, illustrating: (i) G peak intensity (IG at ≈ 1580 cm-1), (ii) 2D peak intensity (I2D at ≈ 2700 cm-1), and (iii) I2D/IG ratio, which demonstrate the multi-layer graphitic nature of the CVD GF without monolayer regions (I2D/IG > 2) (scale bar = 20 *μm*).

GF was synthesized on a nickel (Ni) foam template using chemical vapor deposition (CVD) and characterized to assess its microstructure and graphitic nature (Fig. 2). After synthesis, the Ni foam is etched and cut into 10 mm rounds for experiments (Fig. 2b). Cross-sectional scanning electron microscopy (SEM) analysis revealed two different types of node junctions at the intersections of GF branches. Hourglass-shaped nodes (Fig. 2c(i)) exhibit a thicker sidewall as compared to triangle shaped nodes (Fig. 2c(ii)), with a structure thickness ranging from 5.46 ± 1.78 microns to 3.26 ± 0.91 microns for the hourglass and triangle nodes respectively. Surface SEM further reveals a hierarchical, interconnected porous structure (Fig. 2c(iii)) with a wrinkled topography (Fig. 2c(iv)) resulting from the difference in thermal expansion coefficient between the graphene and underlying Ni template - leading to increased focal adhesion sites for cell attachment^46,51^.

Raman spectroscopy was employed to assess the graphitic nature of the GF across six samples from six different synthesis batches (Fig. 2d). The characteristic G (I_G_ ≈ 1580 cm^−1^) and 2D (I_2D_ ≈ 2700 cm^− 1^) peaks, indicative of graphitic material were consistently observed in all samples. The G peak corresponds to the in-plane vibrations of sp^2^-bonded carbon atoms, while the 2D peak reflects the second-order two-phonon process characteristic of few-layer graphene. The minimal variance in peak positions and intensity ratios across batches demonstrates the reliability of the synthesis process, ensuring batch-to-batch consistency for high-volume studies. Spatial mapping across a 100 μm^2^ area highlighted the ratio of 2D-peak (I_2D_ ≈ 2700 cm^−1^) to G-peak intensity (I_G_ ≈ 1580 cm^− 1^), with an I_2D_/I_G_ peak ratio < 2 indicating multi-layer graphene (Fig. 2e).

### ES Chamber Design, Fabrication, and Characterization

**Figure 3:**
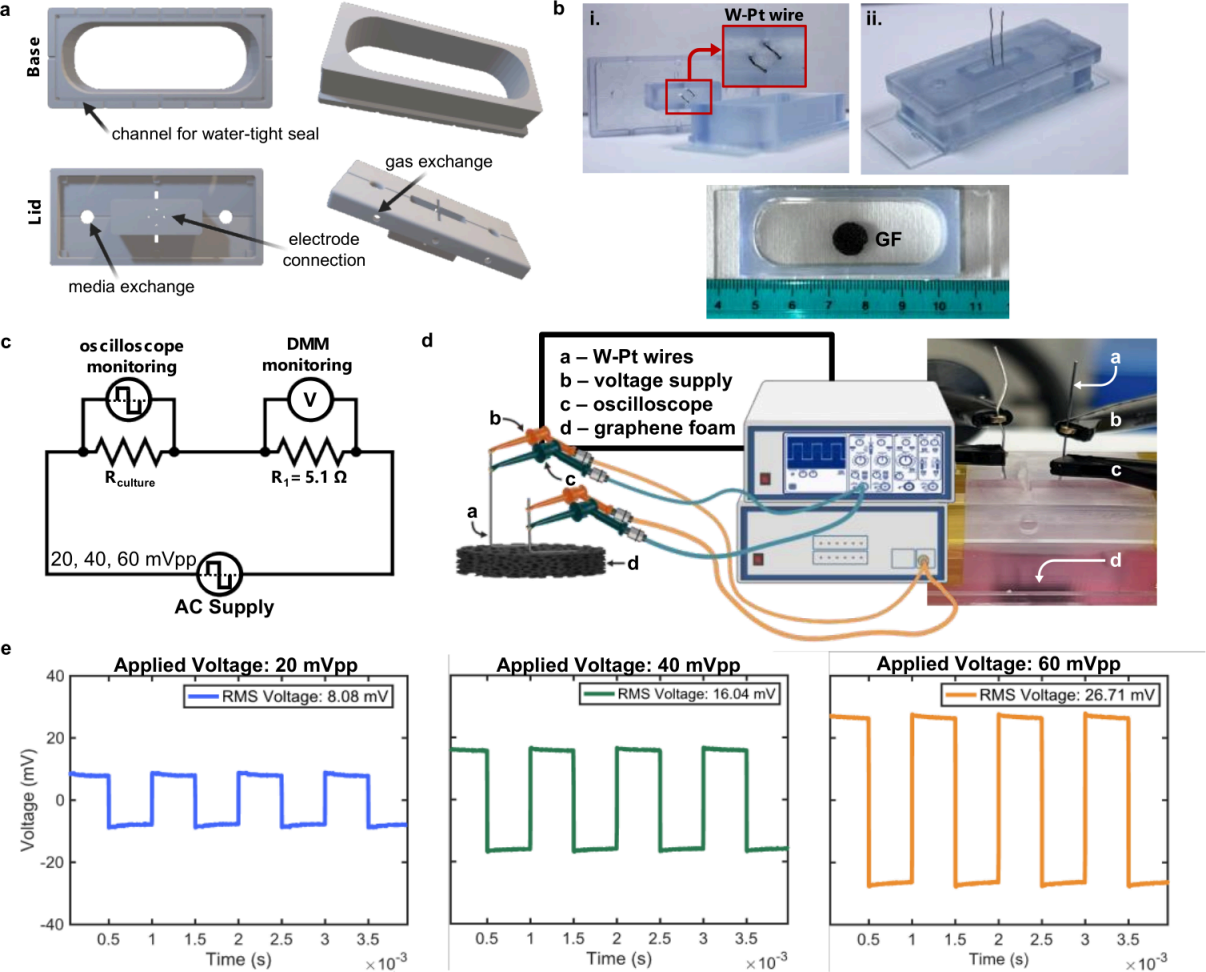
Custom-designed electrical stimulus chambers for direct electrical stimulus *in vitro*. a) Three-dimensional CAD renderings of the DLP printed electrical stimulus chambers. b) Images illustrating various components of the assembled chambers: (i) Platinum coated tungsten wires are secured in the chamber lid for graphene foam connection, (ii) extension of wires beyond the lid for oscilloscope and function generator connection, and (iii) size comparison of the chamber on a glass slide with graphene foam in the center. c) Wiring diagram depicting the setup for waveform monitoring across the graphene foam, including a reference resistor of 5.1 ohms for voltage verification using an oscilloscope and digital multimeter (DMM). d) Schematic representation detailing the chamber connections, including the placement of (a) wires, (b) voltage supply, (c) oscilloscope, and (d) graphene foam. Also shown is a digital image of the connections within a fully assembled device. e) Oscilloscope voltage readings across the graphene foam with applied biphasic square voltage inputs of 20, 40, and 60 mVpp, with an average measured RMS voltage for each input of 8.08 mV, 16.04 mV, and 26.71 mV respectively.

Custom ESCs were fabricated with direct light processing (DLP) 3D printing (Fig. 3a) and designed to ensure that the GF was fully submerged in the media while providing access tungsten (W) leads coated with platinum (Pt) (W-Pt) to connect with the GF, enabling direct stimulus of the cells grown on GF bioscaffolds (Fig. 3b–d). When biphasic square voltage inputs of 20, 40, and 60 mVpp were applied to bare GF submerged in cell culture media the average RMS voltages across the GF were measured at 8.08 ± 1.2 mV, 16.04 ± 2.6 mV, and 26.71 ± 0.93 mV, respectively (Fig. 3e). When the GF was removed from the chamber, no voltage could be read from the oscilloscope due to the high resistance and low conductivity of the media. These results confirm the system’s capability to provide precise and repeatable electrical stimulation when GF is utilized as an active bioscaffold. To address concerns about the potential breakdown of cell culture media during ES we monitored the pH stability of the media before and after 5 minutes of stimulation at each voltage input^52^. The pH remained within the physiological range before and after ES (Figure S1).

The tortuosity factor quantifies the reduction in diffusive transport due to the convoluted flow paths in porous media like GF bioscaffolds. To directly compare the electric fields in our system with published literature, the tortuosity factor for GF bioscaffolds was calculated using the image-based computational tool *TauFactor*, which evaluates transport properties based on microstructural data (Figure S2)^57^.

The analysis yielded a tortuosity factor of 39.3, indicating that transport pathways within the porous GF bioscaffolds are approximately 39.3 times longer than the direct straight-line path in a solid material. Additionally, the directional percolation of conductive material was calculated to be 93.8%, with a phase volume fraction of 6.54%, indicating that the conductive paths are highly interconnected despite the relatively small volume fraction. Using the tortuosity factor, the Pt-W electrode distance of 3.17 mm, and RMS values, effective electric fields were estimated as 0.71 ± 0.02 mV/cm, 1.42 ± 0.06 mV/cm, and 2.19 ± 0.1 mV/cm for 20 mVpp, 40 mVpp, and 60 mVpp input voltages, respectively. These values provide a quantitative basis for understanding the localized field strengths within the scaffold under different ES conditions.

### Architecture and Distribution of ATDC5 Cells Cultured on GF in ESChambers

**Figure 4:**
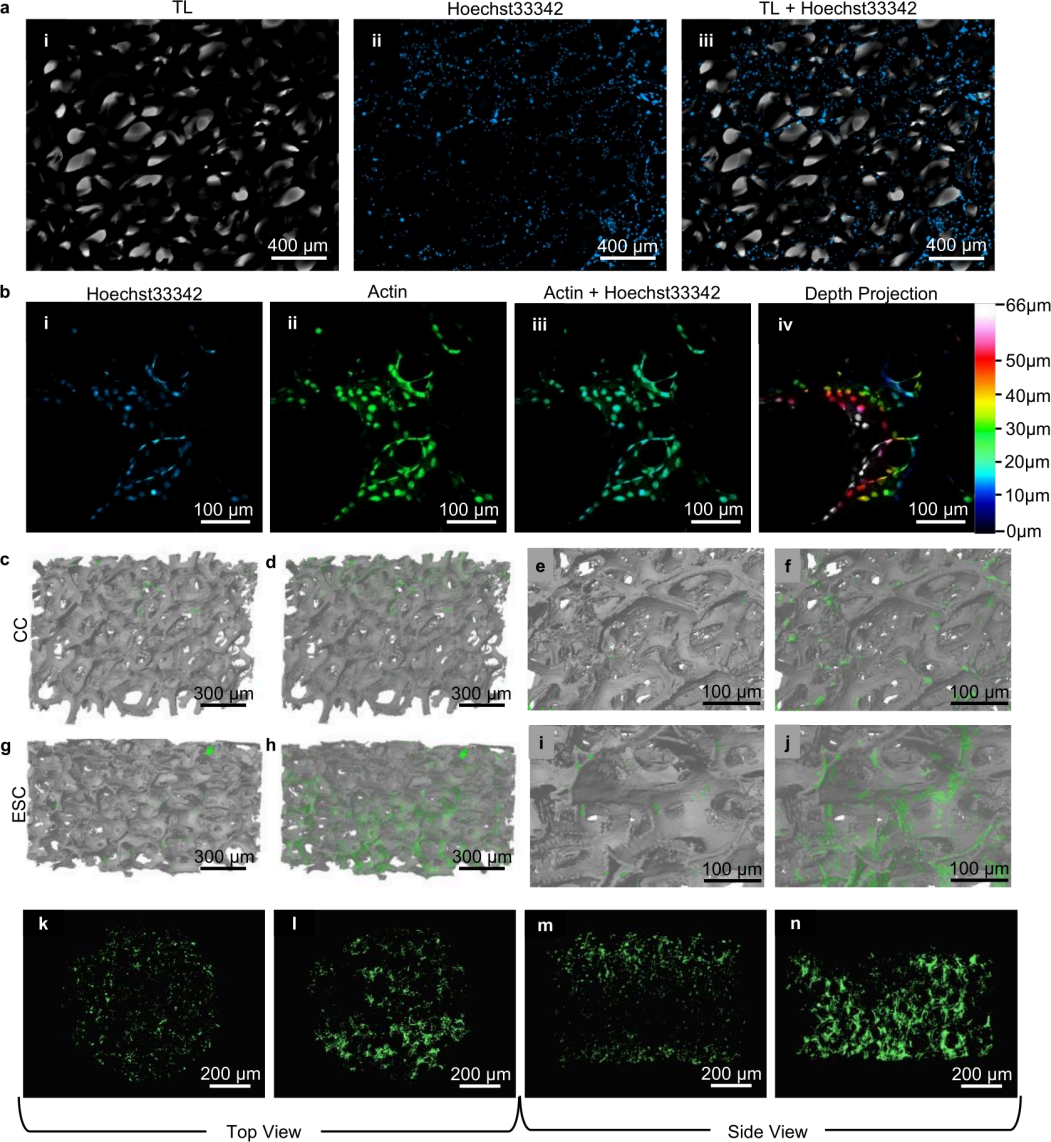
Comparative cellular architecture on GF in ESCs and CCs. a) Cellular imaging on graphene foam: i) brightfield microscopy reveals the scaffold’s structure, ii) Hoechst33342 staining (blue) highlights nuclei distribution, and iii) an overlay of i and ii illustrates nuclei alignment with the scaffold. b) Confocal microscopy showcasing cellular interaction with the scaffold: i) Nuclei (Hoechst33432), ii) Actin fibers labeled with AF488+10 nm colloidal gold, iii) an overlay of i and ii, and iv) color-coded z-stack projection depicts limitations of confocal imaging in 3D opaque GF. c-n) MicroCT imaging contrasts cellular morphology and distribution when cultured in CC versus ESC (without electrical stimulation) displays enhanced cellular structure, density, and migration within GF: c-f) Side and zoomed views of GF-cells cultured in CC with 0% (c & e) and 60 % (d & f) GF opacity show limited cellular elongation and density, g-j) Side and zoomed-in views of GF-cells cultured in ESCs display enhanced cellular elongation and density. k-n) Top and side views of microCT in 3D: CC (k, m) show rounded, sparsely distributed cells, whereas cells in ESC (l, n) exhibit denser, more elongated cells penetrating deeply into the scaffold, aligning with graphene foam structures.

To compare cell attachment and proliferation on GF in ESCs with commercially available culture chambers (CC), we utilized a gold nanoparticle labeling technique developed in previous work^58^. This method enables the characterization of cells on opaque GF using fluorescence microscopy, SEM, and microCT imaging for 3D volumetric analysis. Fluorescence microscopy of cells cultured on GF within the ESCs (Fig. 4a) revealed substantial cell coverage across the intricate GF branch network, as confirmed by Hoechst-labeled nuclei, indicating robust cellular proliferation.

Fluorescently labeled actin highlights cellular structures, allowing for the visualization of cells spanning the pores of GF, and providing insights into cell morphology and growth dynamics. However, confocal z-stacks were limited to a thickness of ~ 66 μm, as evident from the maximum intensity projection (MIP) color-coded representations of z-depth (Fig. 4b(iv)). This limitation underscores the challenges posed by GF’s opacity, thickness, and hollow branches when using standard optical techniques for imaging.

MicroCT analysis of GF samples from both the CC and ESC revealed an average structure thickness of 10.11 ± 2.96 μm with 97.08% porosity and 9.15 ± 3.14 μm with 98.14% porosity, respectively, illustrating the structural consistency across CVD GF bioscaffolds and enabling direct comparison between the two culture environments. Antibody-gold labeling of actin revealed enhanced cellular integration in the ESCs compared to CCs (Fig. 4c–n) and volumetric analysis (Table 1) provided further insights into cellular distribution within each 3D environment. Cells cultured in ESCs exhibited a higher total cell volume (Obj.V/TV), more than double that of the CC (Fig. 4d & h). Additionally, cells grown in ESCs exhibited a surface to volume ratio

(Obj.S/Obj.V) ratio of 841.31 mm^−1^, compared to 596.5 mm^−1^ in control samples.

Further analysis of cell distribution in the GF was conducted by calculating the surface density of cells within the total volume of GF (Obj.S/TV), yielding measurements of 0.304 mm^−1^ for the ESC and 0.092 mm^−1^ for the CC. Rendered 3D models from MicroCT data revealed cells within the GF branches through microcracks at full opacity (Fig. 4e & i). Computationally reducing the opacity to of GF 60% allowed clearer visualization of internal cell morphology, showing more spread and elongated cells in the ESCs compared to the spherical morphology observed in the CCs )Fig. 4f and j).

To evaluate cellular interconnectivity, we calculated the connectivity density (Conn.Dn), which quantifies the density of bi-directionally connected cells within the GF volume, forming a continuous network. The Conn.Dn of cells cultured in ESCs was higher than in control chambers, at 459.78 mm^−3^ compared to 34.86 mm^−3^. The number of distinct cell clusters was 8,838 in ESC and 3,388 in control chambers. After fabrication and verification of performance, ESCs were utilized to provide direct ES to GF seeded with ATDC5 chondrogenic progenitor cells at 20, 40, and 60 mVpp for 7 days followed by an additional 7-day growth period for a total culture time of 14 days.

Dynamic Mechanical Analysis of GF-cell Constructs Following ES`

**Figure 5:**
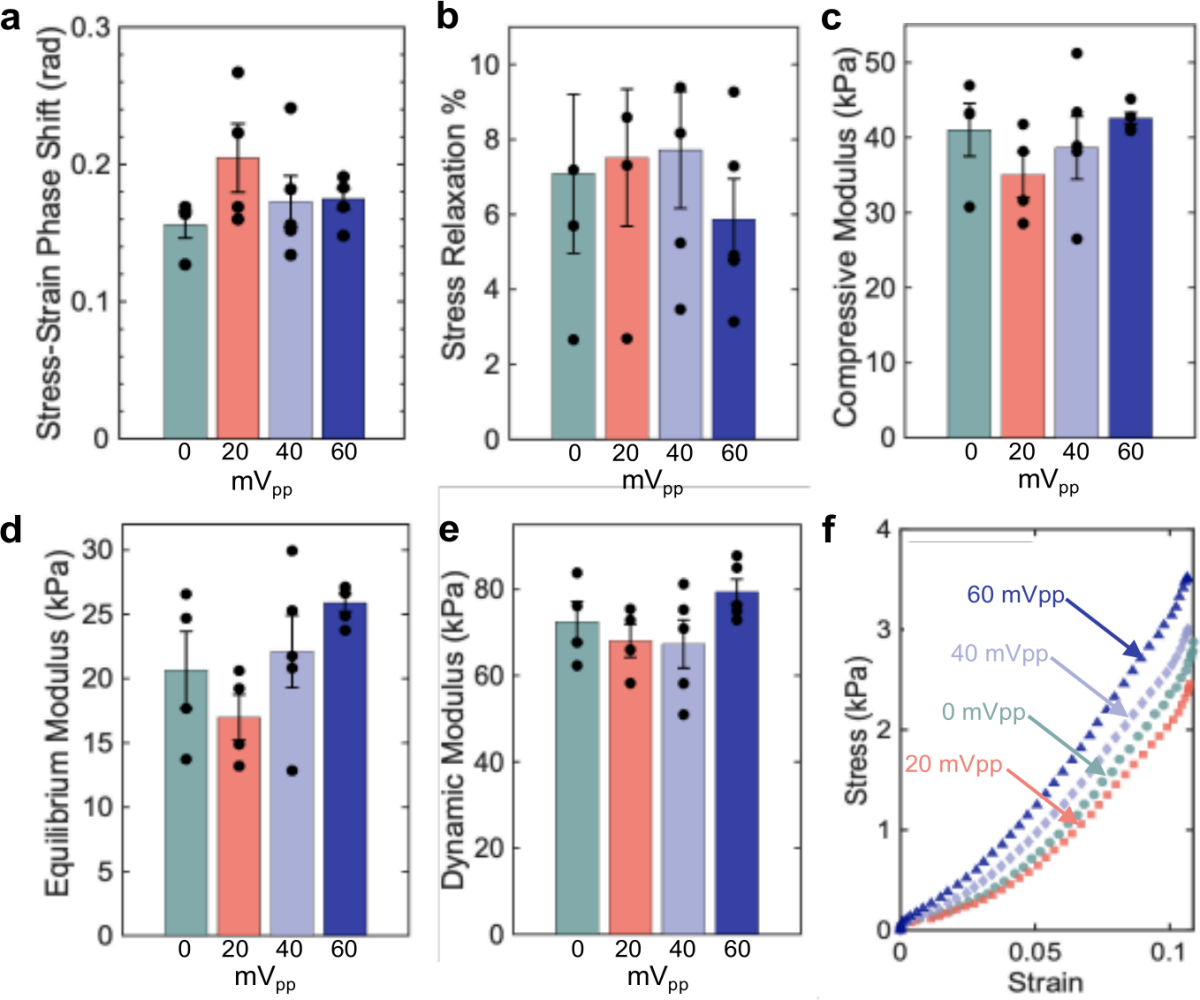
Mechanical characterization of GF-cell composites post-electrical stimulation. Bar plots illustrating the results of dynamic mechanical analysis following 14 days of cell culture with 7 days of direct electrical stimulation applied to ATDC5 cells cultured on GF. a) stress-strain phase shift, b) stress relaxation, c) compressive modulus, d) equilibrium modulus, and e) dynamic modulus, each incorporating data points and standard deviations to reflect variability. f) Stress-strain curves used to derive compressive modulus values, demonstrating the mechanical responses of GF-cell composites across different electrical stimulation conditions.

Following previous work, a DMA protocol was designed to measure changes in stress relaxation, phase shift, compressive linear modulus, equilibrium modulus, and dynamic modulus (Figure S6)^55^. The compressive linear modulus and equilibrium modulus quantify the elastic properties of the GF-cell composite, while the dynamic modulus, phase shift, and stress relaxation assess its viscoelastic properties, which are essential for normal cartilage function^14,59,60^. The DMA results summarized in Fig. 5 illustrate mechanical observations in GF-cell constructs subjected to distinct electrical stimuli compared to control samples (0 mV) cultured in ESCs without stimulation. The tests demonstrate a ∼ 25% increase in equilibrium modulus and a ∼17% decrease in stress relaxation between the 0 mV sample and the 60 mV sample. Notably, the equilibrium modulus decreased by ~52% from the 60 mV samples to the 20 mV samples. The 20 mV samples consistently exhibited inferior mechanical properties and high sample variability characterized by the stress-strain curves and high standard deviations (Fig. 5f).

As commonly observed in cartilage tissue under unconfined compression, the dynamic moduli in all samples for our study were significantly larger than the equilibrium moduli, with a 3-fold increase in the 0 mV, 40 mV, and 60 mV samples, and a 4-fold increase in the 20 mV samples. This is indicative of the viscoelastic behavior seen in natural cartilage, where the response to loading is time-dependent^61^.

**Figure 6:**
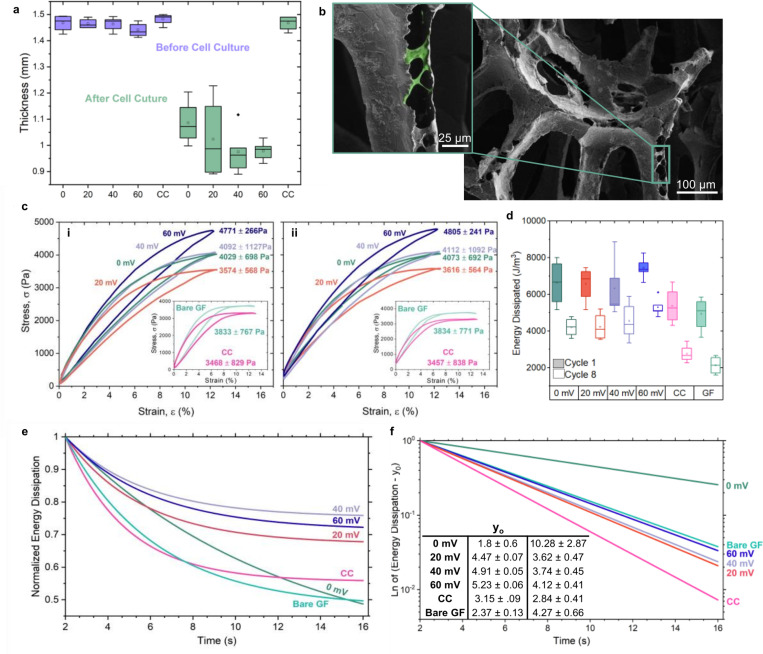
Structural dynamics and cellular interactions in GF-cell constructs, a) Box plots illustrating the significant changes in thickness of before (GF bioscaffolds) and after (GF-cell composites) cell culture. b) SEM image depicting cells bridging structural branch cracks within GF. c) Comparative stress-strain hysteresis loops for the initial (i) and eighth (ii) compression cycles from electrically stimulated samples, supplemented with insets that contrast the mechanical behaviors of cells cultured in control chambers and bare GF. d) Box plots representing energy dissipation across initial and final cycles for samples ES samples, samples from control chambers, and bare GF. e) Energy dissipation over compression cycle time after the first compression (t=2) fit with an exponential decay model. f) Adjusted energy dissipation $\left(y_{adjusted}=\frac{y_{fit}-y_{0}}{A}\right)$ plotted on a log scale to visualize changes in slopes across samples, inset with a table of the ${\text{y}}_{0}$ and τ values.

The thickness of GF samples cultured in ESCs significantly decreased after cell culture, likely due to internal compressive stresses exerted by cells spanning the GF branches, with the 20 mV ES sample exhibiting the highest sample variation (Fig. 6a & b), consistent with the variation seen in the DMA results. The samples cultured in control chambers showed no significant difference in thickness after cell culture which is indicative of the volumetric analysis on cell density and connectivity.

Hysteresis loops of stress versus strain were generated for GF-cell constructs to assess energy dissipation during compression cycles in preconditioning (Fig. 6c). Loops for the first (Fig. 6c–i) and eighth (Fig. 6c–ii) cycles were compared to examine differences in energy dissipation over time. This energy dissipation can be associated with rearrangement of the structure as a response to cyclic loading. By plotting energy loss over time during preconditioning, we evaluated the GF-cell constructs’ ability to adapt to low cyclic loading and assessed whether the constructs became more efficient in handling the applied load over time^62^. For this analysis we compared the behavior of samples cultured in ESCs with and without stimulus to samples cultured in CCs as well as bare GF without cells.

Energy dissipated for the first compression cycle in preconditioning was significantly larger than that of the eighth compression cycle for all samples, as confirmed by paired sample t-tests (Fig. 6d). The energy dissipation differences between bare GF and the 60 mV samples for the first compression cycle were significantly different (p = 0.032) where bare GF exhibited an energy loss of 4.93 ± 0.94 kJ/m^3^ and the 60 mV samples had an energy loss of 7.44 ± 0.59 kJ/m^3^ (Table 2). The maximum stress during compression cycles one and eight was measured from the peak stress value of each hysteresis loop. The 60 mV sample exhibited the highest maximum stress, with values of 4771 ± 266 Pa for cycle one and 4805 ± 241 Pa for cycle eight, demonstrating the consistency of its mechanical properties under prolonged cyclic compression.

The exponential decay analysis of energy dissipation over time revealed significant differences in the behavior of GF-cell constructs where the ${\text{y}}_{0}$ value represents the steady-state energy dissipation level after the transient effects have decayed, and τ indicates the time constant of the decay, reflecting how quickly the system reaches steady-state. The 60 mV samples exhibited the highest steady-state energy dissipation with a ${\text{y}}_{0}$ of 5.23 ± 0.06 kPa, indicating a more robust mechanical response from the constructs with the applied electrical stimulus. In contrast, the 0 mV samples had the lowest ${\text{y}}_{0}$ value, with a steady-state energy dissipation capacity approximately 65.58% lower than that of the 60 mV samples (Fig. 6f).

A higher τ value is indicative of a slower decay to the steady state, reflecting a more prolonged response to cyclic loading. The 0 mV sample exhibited the highest τ at 10.28 ± 2.87 seconds, suggesting a slower adaptation to the applied load. Mechanically, these results imply that the electrically stimulated samples, particularly at 60 mV, exhibit a more stable and sustained mechanical response likely due to improved cellular adaptation and integration within the GF.

## Discussion

Cartilage TE faces a distinct set of challenges, particularly in replicating the intricate microenvironment and mechanical properties of native AC. These complexities necessitate innovative strategies to enhance cell behavior and provide structural integrity to support proper tissue function. This study introduces an approach that combines advanced materials and methodologies to address these challenges. With their unique mechanical properties, GF bioscaffolds present a promising solution for sustained mechanical integrity and chondrogenic differentiation over long-term cultures without requiring additional functionalization. Given the varied outcomes of ES on cell behavior due to differing application methods, GF serves as a model substrate for in-depth studies of ES in cartilage TE. GF electrical properties and 3D structure provide a versatile platform to systematically investigate and optimize the parameters of ES, with a focus on achieving consistent and reproducible enhancements in the mechanical properties of GF-cell composites, potentially driven by chondrogenic differentiation and ECM contributions^63,64^.

Our custom-designed ESCs address key challenges in delivering effective ES for TE, including electrode biocompatibility, media stability, and homogeneity in cell distribution by facilitating scaffold-coupled ES within a 3D environment. ESCs promote cell attachment and nutrient distribution for the duration of the culture period, thereby addressing a challenge identified in preliminary studies when culturing on GF. Additionally, the customizability of the ESCs support rapid prototyping and high experimental throughput, making it an efficient tool for testing the influence of ES.

Volumetric and structural analyses show clear differences in cell morphology, distribution, and integration on GF substrates between ESCs and CCs (Fig. 4). Cells in ESCs exhibited larger volumes and elongated and highly connected morphologies, suggesting that ESCs support cellular communication and promote beneficial cell behavior on hydrophobic GF bioscaffolds (Fig. 4h & j)^65^. In contrast, cells in CCs showed lower volumes, fewer clusters, reduced connectivity, and spherical morphologies (Fig. 4d &f), indicating a less supportive environment for cell attachment and integration.

GF’s hydrophobicity and lightweight structure present challenges for cell culture, often requiring surface coatings that can impair scaffold-coupling, reduce electrical properties, and restrict pore interconnectivity needed for cell infiltration and nutrient transport within the 3D matrix^66^. ESCs overcome these limitations by ensuring effective nutrient diffusion and providing a reliable platform for scaffold-coupled ES that is both repeatable and easily monitored.

Our ESC design establishes a direct contact with the GF substrate, ensuring efficient transmission of electrical signals to the GF-cell interface and overcoming the limitations of traditional ES methods such as capacitive or media-based coupling. Conventional methods often face challenges like thermal heating, media degradation, cytotoxicity, and inconsistent electric field distribution leading to variability in cell behavior^33–35,67^. In contrast, our ESC design provides consistent, reliable ES delivery to GF-cell composites, enhancing mechanical reinforcement through ES while leveraging GF’s superior conductivity and lower resistance compared to traditional scaffold materials and cell culture media. This characteristic is particularly beneficial for applications requiring uniform ES including electroactive cell models for bone, muscle, cardiac, and neural TE applications^44,68–72^.

The DMA results revealed important insights into the mechanical behavior of GF-cell constructs, demonstrating that scaffold-coupled ES enhances mechanical properties. After 14 days of culture, our GF-cell constructs exhibited equilibrium and dynamic moduli of 25.9 ± 1.51 kPa and 79.41 ± 6.57 kPa respectively, under the 60 mV condition. These values show a considerable improvement compared to previously reported results for the GF-ATDC5 system cultured in CCs without ES, where equilibrium and dynamic moduli reached ~ 7.8 kPa and ~ 29 kPa, respectively, after 28 days of culture^55,56,73^. These findings demonstrate that submerged culture conditions alone significantly enhance the mechanical properties of GF-cell constructs with scaffold-coupled ES enabling further fine-tuning of mechanical behavior for more tailored applications.

The equilibrium moduli, reflecting the sustained stiffness stiffness of the GF-cell constructs, were consistently lower than the dynamic moduli indicating viscoelastic behavior. This mirrors native AC, where the dynamic modulus is often more than double the equilibrium modulus, allowing cartilage to stiffen under dynamic loading while maintaining compliance under static conditions^73–75^. The 60 mV condition showed an increase in equilibrium modulus alongside a decrease in stress relaxation, suggesting more robust cellular structures and potential maturation of ECM (Fig. 5b &d)^76–78^. These mechanical trends are consistent with literature highlighting the significant role of load-bearing ECM molecules, such as collagen and aggrecan, in reinforcing scaffold structures, where collagen exhibits exceptional stiffness with a bulk moduli ranging from 8 GPa to 35 GPa under varying pressures^63,79^.

GF-cell constructs cultured in ESCs showed notable thickness changes after cell culture, while those in CCs exhibited minimal variation, consistent with volumetric analysis of cell density and interconnectivity. We propose that cell growth across the pores and branches of the GF led to material compression, as supported by SEM images showing cells within the GF structure (Fig. 6b). These observations underscore the importance of investigating the material structure alongside the mechanical properties, as cell-induced compression within the GF could significantly impact the overall properties and functionality of the bioscaffold.

Under cyclic loading, viscoelastic materials dissipate energy through fluid-solid friction and molecular rearrangements of the solid matrix^80^. Our analysis highlights that energy dissipation behavior, driven by the flow of incompressible water out of the ECM, is significantly enhanced by ES^81^. The 60 mV samples exhibited the highest steady-state energy dissipation, indicating a more robust mechanical response to cyclic loading (Fig. 6e) which is vital to delaying fracture initiation and propagation^82^. In contrast, the 0 mV samples had the lowest steady-state energy dissipation, suggesting a less effective mechanical reinforcement, despite having similar mechanical properties measured with DMA.

**Figure 7:**
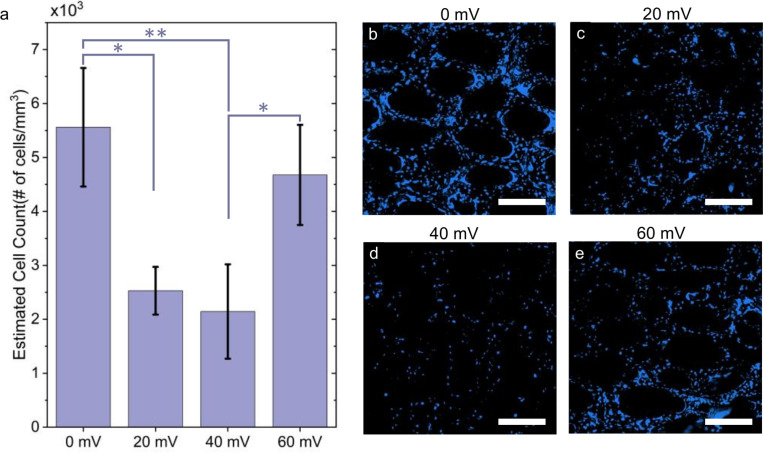
Estimated cell count and representative nuclei staining for each ES condition. (a) Bar graph showing estimated cell count (# of cells/mm3) within GF samples subjected to different ES conditions: 0 mV, 20 mV, 40 mV, and 60 mV. One-way ANOVA analysis indicates a statistically significant effect of ES on cell proliferation (n = 3, F = 10.94, p = 0.0033). Tukey’s post-hoc comparisons reveal significant differences between specific conditions, particularly between 0 mv and both 20 mV and 40 mV conditions, as well as between 60 mV and 40 mV conditions. Representative nuclei Hoechst counterstaining images for each condition: b) 0 mV, c) 20 mV, d) 40 mV, e) 60 mV (b-e scale bar = 300 μm)

An estimated proliferation analysis, though limited to the GF surface, revealed similar cell densities between the 0 mV and 60 mV samples (Fig. 7e) (5.56 ± 1.1 × 10^3^ cells/mm^3^ and 4.68 ± 0.93 × 10^3^ cells/mm^3^, respectively), while the 20 mV and 40 mV condition exhibited significantly (p = 0.0033) lower cell densities (2.53 ± 0.44 × 10^3^ and 2.14 ± 0.87 × 10^3^ cells/mm^3^, respectively). Despite the lower proliferation, the 40 mV samples displayed mechanical properties comparable to the 60 mV condition, suggesting that scaffold-coupled ES at this level may enhance ECM deposition or structural organization. Interestingly, the 20 mV samples, which also had reduced proliferation, showed distinct mechanical trends, including a higher phase shift and decreases in both the compressive and equilibrium moduli compared to all other samples. These observations imply that at lower field strengths, ES may lead to a more compliant and less viscoelastic construct.

The time constant, τ, supports these findings, with the 0 mV sample showing the highest τ, indicating a slower adaptation to cyclic loading (Fig. 5e & f). When comparing energy loss in the eighth cycle, the bare GF had a significantly lower loss than all samples cultured in ESCs, indicating that cellular activity contributes to the mechanical robustness and the ability to absorb and dissipate more energy. The degradation of energy dissipation capabilities in both bare GF and GF-cell constructs cultured in CCs indicate that the constructs might be experiencing changes in their internal structure due to material fatigue, plastic deformation, and micro-cracking^49,80,83^. This implies that the electrically stimulated samples demonstrate a more stable and sustained response, likely due to better cellular adaptation and integration within the GF. This aligns with previous findings that enhanced the mechanical properties of GF through polymer addition, attributed to the filling of voids and defects within the structure^84^. Coupled with the increased cell density in the 60 mV condition, these findings reinforce GF’s potential as a platform for direct scaffold-coupled ES, offering improved control over local field distribution and minimizing limitations associated with capacitive systems, such as field dissipation and high input voltage requirements.

While this study highlights several innovative aspects of GF bioscaffolds for direct ES in cartilage TE, it also identifies areas for future research. Optimizing the mechanical properties to better match native AC and exploring the biological implications of ES on cell function are essential next steps. Expanding this study to include other cell types and implementing comprehensive molecular analyses could provide a more comprehensive understanding of how direct ES influences cellular activity and differentiation. Addressing these limitations could significantly enhance the clinical potential of this approach.

In conclusion, we developed a device for direct scaffold-coupled ES *in vitro* on conductive GF bioscaffolds. Cells cultured in ESCs exhibited a two-fold increase in cell volumes, more elongated morphologies, and a higher connectivity density (459.78 mm^− 3^) compared to CCs (34.86 mm^− 3^, indicating improved cell attachment, proliferation, and nutrient exchange. Mechanical tests revealed the direct ES enhances GF-cell construct properties, with a ~ 25% increase in equilibrium moduli and a 65.58% increase in steady-state energy dissipation capacity measured in samples stimulated at 60 mVpp as compared to controls, suggesting enhanced mechanical reinforcement by the cells. Although ECM was not directly measured, the mechanical improvements under scaffold-coupled ES align with studies showing ECM deposition enhances the elasticity and resistance to deformation in porous scaffolds under mechanical loading^85,86^. Overall, this research establishes a promising framework for integrating conductive bioscaffolds and custom ESCs in cartilage TE, potentially advancing the development of fundamental studies in the effect of scaffold-coupled ES on cell behavior.

## Materials and Methods

### Synthesis of Chemical Vapor Deposition GF

GF bioscaffolds were synthesized in batches on a 1.2 mm thick nickel (Ni) foam template using an open-source chemical vapor deposition furnace fit with a 2-inch quartz tube^87^. Briefly, the Ni template is annealed at 1000°C for 30 minutes, followed by graphene synthesis under a methane (CH_4_) flow at 600 SCCM and 1000°C for 50 minutes before undergoing a cooling cycle down to room temperature (Figure S3). To preserve the structural integrity of the Ni/GF composite, a coating of polymethyl methacrylate (PMMA) was applied and dried before additional post-processing. The coated foam was etched in 3M hydrochloric acid on a hotplate at 40 ºC to aid Ni dissociation and then rinsed with nanopure water. After drying, the PMMA layer is dissolved with acetone before GFs are rinsed and cut into 10 mm rounds with a leather punch.

### Characterization of GF

To visualize the microstructure and surface characteristics of GF bioscaffold the substrates were imaged via scanning electron microscopy (SEM) utilizing a FEI Teneo Field Emission Scanning Electron Microscope. Samples were attached to the SEM post with double-sided carbon tape, and electron micrographs were collected at 10.00 kV and 50 pA. SEM micrographs were used to measure pore size, relative porosity, and structure thickness using FIJI software for comparison with microCT data.^88^ Raman spectroscopy (Horiba Scientific Lab RAM HR Evolution Raman Microscope) was utilized to evaluate the graphitic nature of the GF and to confirm the complete dissociation of the nickel foam template, both spatially and pointwise. 2D/G peak intensity ratios were calculated to further quantify the graphitic nature of CVD GF and to generate spatial maps based on these ratios across a 50 μm by 50 μm sample area. For SEM cross-sectional analysis, as grown graphene foam samples were mounted on a silicon coupon and fixed in place through the addition of liquid polymethyl methacrylate (PMMA), then cured in a furnace at 70°C for 24 hours. A cross-section of the sample was then prepared through manual polishing with a tripod-style sample mount and high-temperature mounting wax. In preparation for SEM, the sample was cleaned by submersion in Acetone for 10 minutes to remove excess PMMA and wax that would result in electron charging. Cross-sectional images were obtained via a FEI-Teneo SEM operating with an Everhart-Thornley Detector (ETD) at 5kV accelerating voltage and 0.20nA current.

### Fabrication and Assembly of Custom ES Chambers

Custom ESC bases and lids were designed using AutoCAD software (Fig. 3a) and fabricated using a Prusa SL1S Direct Light Processing (DLP) 3D printer using Form Labs^™^ Biomed Clear resin. The lids featre corner lifts to facilitate gas exchangle and precisely spaces holes (3.17 mm apat) for wire connections to GF bioscaffolds. Post-printing, the chambers were washed in isopropyl alcohol (IPA) baths to remove excess resin and then cured in a DentMate TRAYDEX^™^ 45 curing oven (385–405 nm) for 10 minutes. The chamber lids were fit with two platinum (Pt)-coated tungsten (W) wires (0.25 mm dia, Thermo Scientific Chemicals) and the chamber bases were secured to glass slides with a biocompatible silicone. The assembly process (Fig. 3b) involved:

1. W-Pt wires secured in the lid to ensure a connection with the GF without compressing the sample.
2. Extension of these wires beyond the lid to connect to an oscilloscope and function generator.
3. Chamber base secured to a glass slide with the GF bioscaffold centered.

### Wiring and Electrical Setup

A wiring diagram (Fig. 3c) details the setup for supplying and monitoring electrical signals across the GF. This setup included a 5.1 Ω reference resistor for verifying voltage and an oscilloscope for monitoring the waveform. The schematic representation in Fig. 3d provides a detailed view of the chamber connections, including the placement of wires, the function generator, oscilloscope, and GF. Additionally, a digital image shows the fully assembled device with the cell culture media and GF in place.

### RMS Voltage Measurements and pH Stability Testing

To confirm that ES did not lead to media breakdown, potentially impacting cell behavior, we measured the pH of the culture media before and after 5 minutes of stimulus. Briefly, bare GF samples were placed in the chamber while the lid with the W-Pt wires was placed atop the sample so that the wires contact the surface of the GF before being secured with a biocompatible polyimide tape. Lids, designed to hold the connection securely, allow for media introduction through a hole in the top to prevent disturbance of the GF-wire connection. A function generator was used to supply biphasic square waveforms at 20 mVpp, 40 mVpp, and 60 mVpp each with an impulse frequency of 1kHz for a duration of 5 minutes (n = 3 per group). The stimulus on bare GF was carried out at 37°C and 5% CO_2_ and the pH was measured before and after stimulus. The measurements showed no significant changes in pH (Figure S1), indicating the system’s ability to maintain media stability during ES. To further evaluate the ES microenvironment an oscilloscope (Tektronix DMM 6500 was used measure the output waveform and calculate the root mean squared (RMS) voltage for each experimental setpoint employed during the pH study. Voltage across the GF at 20, 40, and 60 mVpp, resulted in average RMS voltages of 8.08 ± 1.2 mV, 16.04 ± 2.6 mV, and 26.71 ± 0.93 mV, respectively. An oscilloscope was utilized in all experiments to monitor the ES and verify consistent connections between the GF and W-Pt wires.

#### TauFactor Analysis for Tortuosity and Effective Electric Field Strengths

Tortuosity and effective diffusivity of the GF bioscaffolds were calculated using *TauFactor*, a MATLAB-based computational tool designed for characterizing transport properties in porous media. MicroCT data of bare GF bioscaffolds was acquired at a voxel size of 2.43 μm, and the reconstructed 3D structure was exported as an .stl file for further processing.

The .stl file was loaded into a custom Python script within the PyVista environment, and a central ROI was cropped to ensure an accurate representation of pore density and distribution across the bulk scaffold while limiting extraneous empty space (dimensions: 640 μm × 1260 μm × 490 μm). The ROI was then sliced iteratively along the Z-axis at 10 μm intervals to generate a series of binary images. Each slice was processed using binary thresholding to generate high-contrast images, with scaffold structures in black and voids in white, and saved as a .tiff file for *TauFactor* input (Figure S2a). *TauFactor* simulations were performed using the Dirichlet:Dirichlet method under periodic boundary conditions to model transport properties effectively (Figure S2b). Based on the calculated tortuosity factor and an electrode distance of 3.17 mm, the effective electric field strengths were estimated for input voltages of 20 mVpp, 40 mVpp, and 60 mVpp using the measured RMS voltages.

### Preparing ES Chambers and GF for Cell Culture

Before use for cell culture, assembled ESCs were sterilized in a 70% ethanol bath under sonication for 15 minutes. Prior to cell seeding, the GF bioscaffolds were soaked with 70% ethanol, rinsed with Dulbecco’s phosphate-buffered saline (DPBS), and conditioned in growth media (GM) composed of F12/Dulbecco’s Modified Eagle Medium (DMEM/F12) supplemented with 5% (v/v) fetal bovine serum (FBS) and 1% penicillin/streptomycin for 24 hours.

### Cell Culture

Conditioned GF bioscaffolds were transferred to a well plate before seeding with 1×10^5^ ATDC5 chondrocyte progenitor cells (Sigma Aldrich, St. Louis, Mo, U.S.A) using the ‘drop on’ method and pipetting the cell suspension directly to the topside of the GF substrate^6^. Following a 24-hour attachment period, GF-cell constructs were relocated to the ESCs before securing the lid and wire attachment to the top of the GF bioscaffold, where 3 mL of growth media was added to each ESC (n = 20). ATDC5 cells underwent direct ES at 20, 40, and 60 mVpp using biphasic square wave impulses with a frequency of 1 kHz for 5 minutes daily, for 7 days, followed by an unstimulated growth for an additional 7 days, resulting in a total growth period of 14 days. Additional samples were harvested after a 48-hour attachment period to assess cell viability via live/dead staining (live: NucBlue^™^ Live ReadyProbes^™^ Reagent (Hoechst 33342) /dead: NucRed^™^ Live 647 ReadyProbes^™^ Reagent)(Figure S5). Control samples (0 mV) were incubated for 14 days in ESCs without ES and negative controls (n = 5) were incubated in CCs (Fisher Scientific). During the growth phase, cells were incubated in GM at 37°C with 5% CO_2_. Cell proliferation was monitored using transmitted light microscopy, and GM was replenished daily, including immediately after each electrical stimulation session, for the entire growth period.

### Immunostaining

For fluorescence, SEM, and microCT comparisons between the CCs and ESCs, additional samples were cultured for a 7-day period on GF in each chamber type, fixed with 0.2% paraformaldehyde, permeabilized with 0.1% Triton-X and directly labeled with β-Actin polyclonal antibody (Thermo Fisher Scientific) at a concentration of 1 μg/mL before incubation at 37°C for 30 min. GF-cell constructs were rinsed 10 times with PBS diluted in nanopure water (10:1), then stained with a 30 μg/mL concentration of Goat anti-Rabbit IgG (H + L) Secondary Antibody Alexa Fluor 488 − 10 nm colloidal gold, incubated at 20°C in the dark for 30 min, and then rinsed 10 times in diluted PBS and dried. The diluted PBS rinsing steps ensure that salt crystals that form from drying do not affect SEM or microCT acquisition. Samples were then counterstained with NucBlue (Hoechst 33342, Thermo Fisher) before imaging with a Zeiss LSM900 confocal system combined with a Zeiss Axio Observer.Z1. Brightfield and Hoechst33342 overlaid images were acquired using the EC Plan Neofluar 5x/0.16 M27 objective and an excitation wavelength of 353 DAPI emission. Confocal Z-stack micrographs were acquired using the Plan-Apochromat 10x/0.45 objective with laser wavelengths of 405 and 488 nm at a laser power of 0.6% and a 19 μm pinhole. Fluorescent image processing was performed with ZEN 3.8 software. Widefield imaging of Hoechst stained nuclei for each experimental condition in ESC chambers (n = 3) was performed using an Agilent Cytation 10 cell imaging multi-mode reader with a 10× Plan Fluor objective and an excitation wavelength of 377 nm (Figure S4). Cell counts were obtained with the Biotek Gen5 Software (Agilent) cell counter. Nuclei were counted based on size (5–30 μm), with thresholding applied to separate overlapping objects. Cell density was estimated by multiplying the Z-thickness of each image by the field of view area and dividing the total cell count by the calculated volume.

### Microcomputed Tomography

Comparisons of the cell culture environment between ESCs and CCs were performed with microCT and 3D volumetric analysis (SkyScan 1172 X-ray microCT). Briefly, gold labeled GF-cell samples were mounted onto a porous polyethylene pipet filter with double-sided tape, where a drop of 70% ethanol was placed atop the GF to ensure mounting to the tape without needing to add pressure to the top of the sample. After drying, the GF-cell / filter was placed upright on the sample holder and secured into place with double-sided tape to eliminate scan artifacts due to random movement^89–91^. Scan acquisition GF-cell constructs was conducted with a 26 kV source voltage, 145 μA current, 4-Watt power, 4k resolution, and 2650 ms exposure time. Scan parameters were defined with a rotation step size of 0.25°, ten-frame averaging, and 2.43 μm pixel size. NRecon software was used to reconstruct the angular projections into cross-sectional slices for 3D reconstruction and volumetric analysis with an attenuation value for all samples from 0 to 0.312. Bruker SkyScan CT Analyzer (CTan) software was used to binarize the 2D images; the GF was binarized with a threshold value of 25 − 115 range on the contrast scale, while gold labeled cells were segmented to a threshold value of 115–255, allowing for individual 3D reconstruction and analysis of both the GF bioscaffold and cells within the scaffold structure. GF structure thickness and porosity, and cell Obj.V/TV (%), Obj.S/Obj.V (mm-1), Obj.S/TV (mm-1), Conn.Dn, and number of objects were calculated from the segmented scans with CTan software. 3D reconstruction of the GF environment was qualitatively analyzed using CTVol software, where cell reconstructions were overlayed with GF and false colored to green with the transfer function editor using the linear interpolation method to highlight the cell architecture versus the GF structure.

### Dynamic Mechanical Analysis

Mechanical tests utilizing unconfined compression were performed using a bioreactor on the 14th day of cell culture for samples stimulated at (n = 5) (Figure Sa). The bioreactor consisted of a tissue chamber and a compressive assembly that used a high precision voice coil actuator (SMAC, Carlsbad, CA) fitted with a 1 N-load cell (FUTEK Inc., Irvine, CA, resolution: 5 μN) for force reading. For all experimental conditions, GF-cell constructs were taken directly out of media and tested immediately. For each experimental condition, GF-cell constructs were taken directly out of media and tested immediately. As geometry plays a pivotal role in dynamic mechanical analysis, a protocol was developed to precisely measure the thickness of GF-cell constructs and dynamic testing parameters were optimized for examining the mechanical properties of GF bioscaffolds. After the cell culture period, the GF-cell constructs were taken out of the ESC and placed in the tissue chamber of the bioreactor. A 0.02–0.03 N preload was then applied to each GF-cell construct to measure its thickness and ensure effective contact before DMA is performed.

The DMA testing method consists of sinusoidal cyclic preconditioning to 14% peak strain at 0.5 Hz with 5 seconds of rest following the precondition, ramp loading to 12% strain, 120 seconds of relaxation, followed by dynamic compressive loading at 1% amplitude with a strain velocity of 10%/second (Figure S6b). Dynamic loading conditions are consistent with previous studies testing the same materials^55,56^. Following DMA, compressive modulus, equilibrium modulus, stress relaxation %, dynamic modulus, and phase shift was analyzed across samples where Figure S6b highlights where the measurements took place. Briefly, compressive moduli were determined by calculating the maximum derivative of the stress/strain curve (dSdE) obtained during the last compressive strain in preloading and performing a linear fit of the 12 data points surrounding the peak dSdE value (Figure S6c). Equilibrium modulus was obtained by dividing the stress by strain after equilibrium had been reached, stress relaxation % was measured by comparing the maximum stress reached directly after the initial compression to the stress measured after equilibrium had been reached, dynamic moduli was measured by fitting the last three compressions in the dynamic portion of the test and dividing the amplitude of stress by amplitude of strain, and phase shift was determined by subtracting the fitted phase parameters from the stress-time and strain-time data^55,92^.

### Energy Dissipation Calculations

Force and displacement data for the DMA tests were extracted from the bioreactor software and converted to stress and strain for energy calculations. Engineering stress and strain extracted during the preconditioning protocol were plotted using MATLAB R2022b (MATLAB R2022b, MathWorks, Inc., Natick, Massachusetts, USA), producing a hysteresis loop of the loading and unloading profile for each compression cycle. For each cycle, the area between the arcs was assumed to be the total energy dissipated for that cycle whereas the area below the loop indicated in Figure S6d as strain energy, represents the total energy in a cycle. Hysteresis loop areas and the areas below each loop were approximated using the trapezoidal numerical integration technique in OriginPro (2020b), with the minimum and maximum force values for each loop set as the area constraints.

Energy dissipation over time was fit with an exponential decay model (Eq. 1) and normalized to the maximum stress value after the first compression cycle for each sample set individually. The R^2^ values, root mean square error (RMSE), F statistic, and p-value were calculated for each model to provide a measure of the average deviation between the observed and fitted values as well as a goodness of fit for the model. RMSE values were found to be less than 0.001 for all experimental conditions, indicating that the exponential decay model provides an excellent fit to the data (Table S1). To visualize the difference in slopes for each of the sample sets, the fits were adjusted to ${\text{y}}_{0}$ (kPa), normalized (Eq. 2), and plotted on a log scale (Eq. 3) (Fig. 6e & f).


1, $$y_{fit}=Ae^{-t/\tau }+y_{0}$$

2, $$y_{normalized}=y_{fit}/A$$

3, $$y_{adjusted}=\frac{y_{fit}-y_{0}}{A}$$

Where t is time in seconds, A is the initial amplitude of energy dissipation, τ is the decay constant, and $y_{0}$ is the estimation of the energy dissipation level that the system would reach if compression continued to a steady state.

### Statistical Analysis

Statistical analyses were conducted to evaluate the significance of differences observed across various experimental conditions. One-Way ANOVA and power analysis was used to determine statistical significance in DMA analysis, maximum energy dissipation, and proliferation analysis across samples with an alpha level of 0.05. Paired sample t-tests were used to compare the hysteresis loop areas between the first and eighth compression cycles. The exponential decay fits were statistically evaluated using root mean squared error (RMSE) measurements between the fit and the observations. Statistical significance was determined at an alpha level of 0.05, and all analyses were performed using OriginPro software to ensure robust and accurate interpretation of the experimental results.

## Supplementary Material

Supplement 1

## Figures and Tables

**Table 1:** Comparative volumetric analysis of cellular architectures in GF bioscaffolds cultured in ESC and CC conditions.

	Obj.V/TV (%)	Obj.S/Obj.V (mm^− 1^)	Obj.S/TV (mm^− 1^)	Conn.Dn	# of objects
**ESC**	0.036	841.31	0.304	459.78	8838
**CC**	0.015	596.5	0.092	34.86	3388

**Table 2:** Energy dissipation for ES samples, control samples, and bare GF for the first and eighth compression cycles.

	Energy Dissipation (kJ/m^3^)	
	Compression Cycle 1	Compression Cycle 8
60 mV	7.44 ± 0.59	5.30 ± 0.51
40 mV	6.32 ± 1.58	4.55 ± 1.05
20 mV	6.57 ± 1.00	4.23 ± 0.77
0 mV	6.63 ± 1.27	4.21 ± 0.52
Control	5.34 ± 1.01	2.76 ± 0.49
Bare GF	4.93 ± 0.94	2.13 ± 0.50

## Data Availability

All relevant data supporting the key findings of this study are available within the paper, its Supplementary Information files, and the Source Data file.

## Abbreviations

OA: Osteoarthritis
TE: Tissue Engineering
AC: Articular Cartilage
ECM: Extracellular Matrix
ES: Electrical Stimulus
GF: Graphene Foam
CVD: Chemical Vapor Deposition
DMA: Dynamic Mechanical Analysis
ESC: Electrical Stimulus Chambers
CC: Commercial Chambers
