# Supplementary material for: Direct Scaffold-Coupled Electrical Stimulation of Chondrogenic Progenitor Cells through Graphene Foam Bioscaffolds to Control Mechanical Properties of Graphene Foam – Cell Composites
Source: Res Sq. 2024 Dec 24:rs.3.rs-5589589. Preprint. [Version 1] doi: 10.21203/rs.3.rs-5589589/v1 (PMC11703340; doi:10.21203/rs.3.rs-5589589/v1)
Supplement: Supplement 1 [file NIHPPRS5589589v1-supplement-1.pdf]

Supplementary Files

This is a list of supplementary files associated with this preprint. Click to download.

- SawyerMonetESManuscriptSupplementalRevised.docx
